# Symptoms of Sarcopenia and Physical Fitness through the Senior Fitness Test

**DOI:** 10.3390/ijerph20032711

**Published:** 2023-02-03

**Authors:** Alex Barreto de Lima, Fátima Baptista, Duarte Henrinques-Neto, André de Araújo Pinto, Elvio Rúbio Gouveia

**Affiliations:** 1 CIPER, Faculdade de Motricidade Humana, Universidade de Lisboa, 1499-002 Cruz-Quebrada, Portugal; 2 Course of Physical Education, Universidade do Estado do Amazonas, Manaus 69065-001, AM, Brazil; 3 Research Center in Sports Sciences, Health Sciences and Human Development, Maia University, 4475-690 Maia, Portugal; 4 School of Higher Education, Polytechnic Institute of Porto, 4200-465 Porto, Portugal; 5 Department of Physical Education, Universidade Estadual de Roraima, UERR, Boa Vista 69306-530, RR, Brazil; 6 Department of Physical Education and Sport, University of Madeira, 9000-072 Funchal, Portugal; 7 LARSYS, Interactive Technologies Institute, 9020-105 Funchal, Portugal

**Keywords:** physical fitness, SARC-F, sarcopenia, senior fitness test

## Abstract

Introduction: Physical fitness concerns a set of attributes related to the ability to perform physical activity that may justify the symptoms reported by the elderly in the context of sarcopenia. Objective: This study aimed to investigate the relationship between the perception (symptomatology) of physical functioning (what the person thinks they are capable of) and the capacity itself for physical functioning in elderly people in northern Brazil. Methods: Cross-sectional study that analyzed 312 elderly people (72.6 ± 7.8 years) from the city of Novo Aripuanã, Amazonas, Brazil. Sarcopenia symptomatology was assessed using the SARC-F, a 5-item questionnaire designed for screening sarcopenia in older individuals in five domains: strength, walking aids, difficulty getting up from a chair, difficulty climbing stairs, and falls. Physical fitness was assessed by the Senior Fitness Test (SFT) battery including balance evaluated with the short version of the Fullerton Advanced Balance scale (FAB). Results: ROC curve analysis revealed that the tests with the greatest ability to discriminate participants with significant symptoms for sarcopenia (≥4 points on SARC-F) were arm curl and 6 min walk: the probability of suspected sarcopenia increased exponentially with an arm curl < 11.5 reps for men (se = 71%; sp = 69%; AUC = 0.706, 95% CI: 0.612–0.788; *p* = 0.013) and women (se = 81%; sp = 51%; AUC = 0.671, 95% CI: 0.601–0.735; *p* ≤ 0.001) or with a 6-min walk <408.5 m for men (se = 71%; sp = 63%; AUC = 0.720, 95% CI: 0.628–0.690; *p* = 0.001) and <366.0 m for women (se = 69%; sp = 58%; AUC = 0.692, 95% CI: 0.623–0.755; *p* = 0.0001). Conclusions: Physical fitness assessed through the senior fitness test, particularly the 30-s-arm curl test and the 6-min walk test, can discriminate for suspected symptoms of sarcopenia.

## 1. Introduction

Sarcopenia has been defined as a generalized disease characterized by decreased muscle mass and muscle function [1,2]. As in other diseases, the prevalence of sarcopenia increases with the aging of the population, constituting a public health problem of great priority in the elderly [3]. Although sarcopenia is identified in young people with particular clinical conditions [4] and healthy young people [5], it is in the elderly that sarcopenia has mostly been investigated [6]. The disease varies in severity and can limit the individual’s daily living activities (ADLs) and increase the risk of frailty, hospitalization, functional dependence, and mortality [7]. Most cases of sarcopenia are attributed to physical inactivity and inadequate protein/energy intake [8], although other causes may also contribute [7,8,9,10].

A wide variety of tools are available for screening, evaluating, and monitoring sarcopenia, but population differences in body composition, physical capacity, and perceptions of physical functioning as well as diverse research scenarios have hampered the systematic implementation of these tools [6]. In this context, the vast majority of cases of sarcopenia are not diagnosed [7]. A case-finding approach is recommended practice [2] and the screening of sarcopenia with user-friendly, simple tools is necessary [10]. This approach involves investigating sarcopenia when relevant symptoms are reported [11]. The symptoms/signs that have been most associated with sarcopenia include a history of falls and difficulties in lifting and carrying a shopping bag-like load (4.5 kg), moving around a room, getting up from a chair/bed, or going up a flight of stairs [11]. The SARC-F is the most widely used questionnaire for the rapid screening of sarcopenia [12]. For this purpose, the SARC-F consists of five questions referring to difficulties or events (falls) resulting from muscle weakness [13]. The sensitivity of SARC-F for screening positive cases has, however, been shown to be low in contrast to the specificity, which is high [3,14,15], meaning that SARC-F better signals people who do not have sarcopenia than people who have [2,16]. For this reason, several changes have been investigated including the addition of information to the original SARC-F [3,14]. However, attention is drawn to the fact that most of the answers to the SARC-F questions are due to musculoskeletal fitness and multisensory integration (balance) at the level of the lower limbs to ensure mobility for carrying out activities of daily living (ADLs) [17], while the identification of sarcopenia is assessed using a maximal handgrip strength test (upper limbs) [2]. As people get older, their level of physical fitness decreases [18], compromising, in the first instance, their health and, in the second instance, intrinsic capacity [19].

The Senior Fitness Test (SFT) is a battery widely used to assess the physical fitness of older people in a community context [20]. The SFT is composed of several tests that aim to inform about aerobic, musculoskeletal, and neuro-motor fitness [21], and ultimately about health and intrinsic capacity [22,23,24]. Bearing in mind that the symptoms of sarcopenia are expressed by difficulties in performing activities of daily living due to insufficient physical fitness and a previous history of falls, it was intended to analyze associations between the perception of symptoms as a whole and individually and physical fitness assessed by the Senior Fitness Test. Since this battery of simple and inexpensive tests is widely used in community exercise programs, the question arises as to its relevance for a more objective screening (suspect) of sarcopenia. The purpose of this investigation was to analyze the relationships between the perception of physical functioning (what the person thinks they are capable of) and the capacity itself for physical functioning in elderly people in northern Brazil.

## 2. Methods

### 2.1. Sample and Study Design

The sample included 312 older adults from the community of Novo Aripuanã (Amazonas, Brazil). Of the 942 older adults who met the search criteria, 630 were excluded (215 not meeting the inclusion criteria and 415 declined to participate). Participants were recruited in basic health units, parks, squares, churches, and other public places in the city’s urban area, in addition to invitations broadcast on local radio stations. Participants living in rural areas were excluded from the study due to difficulties in accessing the evaluation site (distance and means of transportation needed). After explanations about the procedures and risks of the study, all participants signed the informed consent form. All assessments were performed at UEA. The following criteria were considered for participant inclusion: (1) older aged 60 and over residing in the community; (2) be independent in carrying out activities of daily living; (3) moderate or high level of cognitive functioning; (4) no contraindications for physical exertion (stroke, neurological diseases, unstable chronic conditions); and (5) without chest pain, and/or angina pectoris and limiting joint pain [25]. The cognitive level was evaluated with the Mini-Mental State Examination (MMSE) [26]. MMSE ≤ 15/30 points were used to exclude the participants of the study.

This cross-sectional study was approved by the Ethics Committee of the Declaration of Helsinki and Resolution 466/12 of the National Health Council, making part of the research project: “Sarcopenic Syndrome—Physical Function, Phenotype and Quality of Life in Elderly with and without Sedentary Lifestyle” (CAAE 74055517.9.0000.5016/Referee 2.281.400).

### 2.2. Instruments

#### 2.2.1. Anthropometric Measurements

Body mass was measured using a calibrated mechanical anthropometric scale (110 CH, Welmy, São Paulo City, Brazil), with participants barefoot and wearing light clothes. Body height was measured using the anthropometric scale metal stadiometer, with participants in an upright position, arms hanging at their sides, heels together, and occipital and gluteal regions touching the upright ruler of the scale. Body mass index (BMI) was calculated by the ratio between body mass and height (meters) squared (body mass/height^2^).

#### 2.2.2. Symptomatology of Sarcopenia

The SARC-F is a 5-item questionnaire designed for screening sarcopenia in older individuals and addresses five domains: strength, walking aids, difficulty getting up from a chair, difficulty climbing stairs, and falls [13]. Each domain has a question, and the answer is scored from 0 to 2 points for each item [13]. The total score ranges from 0 to 10, with ≥4 points indicating a risk of sarcopenia [13]. The (Brazilian) Portuguese-translated version [27] of the SARC-F questionnaire was applied.

#### 2.2.3. Senior Fitness Test (SFT)

According to Rikli and Jones [28], the Senior Fitness Test (SFT) was developed for adults over 60 years of age. It is primarily used to evaluate physical function in healthy elderly people but is also used for people with dementia [29]. The SFT includes six tests: the 30-s Chair Stand Test (CST), the 30-s arm curl test (ACT), the chair sit and reach test, the back-scratch test (BST), the 8-foot up-and-go test (FUG), and the 6-min walk test (6MWT).

#### 2.2.4. Body Balance

Balance was assessed using the short version of the Fullerton Advanced Balance scale (FAB) [30]. The FAB is an assessment tool used to measure the multiple dimensions of balance in older adults. The short version is composed of four tests, each test is scored using a 4-point ordinal scale (0–4), resulting in a maximum score of 16 possible points, representing the optimal balance performance. The cutoff point is 9 out of 16 points, concluding that an elderly person with a score < 9 on the FAB short version scale will be considered at a higher risk of falling [31].

### 2.3. Statistics

Statistical analyses were performed using SPSS (v26.0, Chicago, IL, USA). Descriptive statistics were calculated for all outcome measurements. Comparisons between sex were made by using the Student’s *t*-test. When the assumptions of the parametric tests were not verified, the Mann–Whitney test was used. Given the existence of an interaction effect for sex (*p* < 0.01), logistic regression analysis was used to examine the associations, for each sex, between the physical fitness tests and the risk of sarcopenia assessed by the SARC-F. The odds ratio of the physical fitness tests for predicting the sarcopenia symptoms was also estimated, according to sex, using the logistic regression. Significance was set at *p* < 0.05.

## 3. Results

Table 1 presents the sample characteristics for the total sample and by sex. Men were taller and heavier than women (*p* ≤ 0.001) but there were no differences in the BMI. Regarding the physical fitness tests, males showed better scores on the ACT, FUG, and 6MWT tests compared to females (*p* < 0.05). Conversely, females showed higher scores on BST and FAB. Despite a tendency of women to present a higher prevalence of significant symptoms (≥4 pts), no differences were observed between men and women in terms of total symptomatology.

Table 2 presents the prevalence of each symptom of sarcopenia separately. Symptom 1 relates to strength, symptom 2 to assistance in walking, symptom 3 to rise from a chair, symptom 4 to climbing stairs, and symptom 5 to the occurrence of falls. Difficulty climbing stairs and assistance in walking were the most and least prevalent symptoms, respectively, in both men and women. Table 2 shows a trend toward a higher prevalence of total symptomatology, but not individual symptoms, suggestive of sarcopenia in women compared to men.

Table 3 presents the results of the logistic regression to predict the likelihood of the occurrence of significant symptoms of sarcopenia (≥4 points) according to several attributes of physical fitness evaluated through the SFT. In women (back stretch, up-and-go, balance), in men (chair stand, sit and reach), or in both sexes (arm curl, 6 min walk), all tests showed the ability to discriminate participants with and without significant symptoms for sarcopenia.

**Figure 1:**
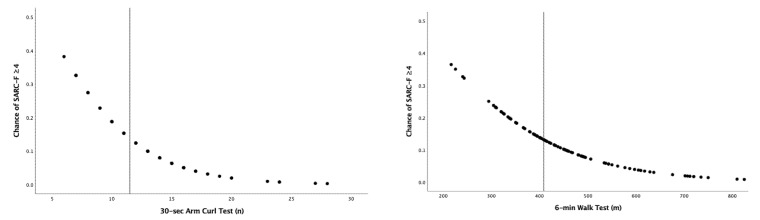

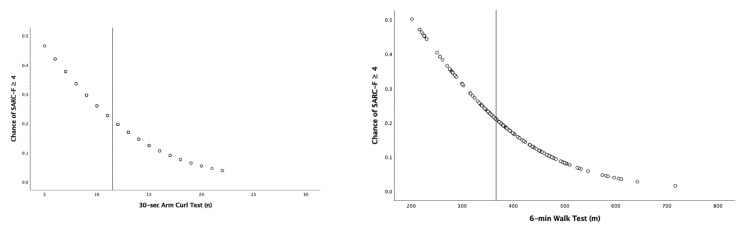
Probability of SARC-F ≥4 points according to the 30-s arm curl test and 6-min walk test (men, black dots (**top**); women white dots (**bottom**)).

Logistic regression analysis and the ROC curve indicated that the likelihood of suspected sarcopenia (associated with SARC-F ≥ 4 points) increased exponentially with an arm curl test < 11.5 reps for men (sensitivity = 71.43%; specificity = 69.39%; AUC = 0.706, 95% CI: 0.612–0.788; *p* = 0.013) and women (sensitivity = 80.95%; specificity = 50.63%; AUC = 0.671, 95% CI: 0.601–0.735; *p* = 0.0001) or with a 6-min walk test <408.5 m for men (sensitivity = 71.43%; specificity = 63.27%; AUC = 0.720, 95% CI: 0.628–0.690; *p* = 0.001 and <366 m for women (sensitivity = 69.05%; specificity = 58.23%; AUC = 0.692, 95% CI: 0.623–0.755; *p* = 0.0001), respectively (Figure 1). The odds ratio of having a SARC-F ≥4 pts decreased by 21.2% in men and 17.1% in women for each repetition (Table 3). Regarding the 6-min walk, the odds ratio of having a SARC-F ≥ 4 pts decreased by 0.7% in men and 0.8% in women per meter walked (or 7–8% per 10 m). 

Table 4 shows the same type of analysis as Table 3, but individually considering each of the symptoms included in the SARC-F questionnaire. In women, a predictive capacity of the shoulder flexibility for the ability to lift and carry a load of 4.5 kg, the strength of arms to get up from a chair, and the balance for the occurrence of falls were observed. In men, no predictive ability of physical fitness was observed for individual symptoms.

## 4. Discussion

This study with elderly people in northern Brazil aimed to analyze associations between symptoms of sarcopenia resulting from physical fitness and reported through the SARC-F questionnaire, and physical fitness itself assessed through the SFT. Specifically, it was intended with this work to know (a) which components of physical fitness assessed through the SFT could screen the symptoms associated with sarcopenia and (b) which values of these components should be considered sufficient, that is, indicators of the absence of significant symptoms of sarcopenia when evaluated by SARC-F. The results revealed a trend toward a higher prevalence of total symptomatology, but not of individual symptoms, suggestive of sarcopenia in women compared to men. Individually, difficulty climbing stairs was the most reported symptom by both men (43.7%) and women (42.5%), while gait difficulty was the least reported symptom by both men (17%) and women (22%); that is, greater symptomatology in line with the physical demands of the activity. In men, falls were the second most reported symptom/event (34.8%), followed by strength to carry a load (29.5%) and to get up from a chair (25%). In women, strength to carry a load (33.5%) and to get up from a chair (29%) were the second and third most reported symptoms, followed by a history of falls (25%).

All physical fitness assessment tests were able to discriminate sarcopenia symptoms, although some tests were able to predict the presence of significant symptoms only in men and others in women. showed the ability to discriminate for the symptomatology of sarcopenia, although some tests were more predictive in men and others in women. The 30-s arm curl and the 6-min walk are noteworthy as they are tests with the greatest ability (acceptable discrimination) to suspect sarcopenia in both sexes. The increase of 1 repetition in the 30-s arm curl test corresponded to a decrease in the odds ratio of suspicion of sarcopenia of 22% in men and 17% in women. With the increase in the distance covered in the 6-min walk test, a decrease in the odds ratio of sarcopenia suspicion was also observed in both sexes: the decrease was 7–8% for every 10 m of distance covered.

Interestingly, the cutoff values of these tests for suspected sarcopenia coincided with the cutoff values proposed by Rikli and Jones [32] to distinguish between maintenance and the risk of loss of functional independence in older adults (11 reps in arm curl and 366 m in 6 min walk). This means that the SFT, usually implemented in community programs to assess physical fitness and identify the risk of loss of functional independence, also seems to show capacity for screening (suspect) sarcopenia. Additionally, this study also showed that the reference values for screening for sarcopenia appear to be similar to the screening values for the risk of loss of functional independence, at least concerning the 30-s arm curl test and the 6-min walk test. If the most prevalent sarcopenia symptoms are related to difficulty climbing stairs and carrying loads, it is likely that the physical fitness components that most discriminated sarcopenia symptoms in our sample were the 6-min walk test (the SFT does not assess stair climbing) and the 30-s arm curl test. Physical fitness is the ability to perform daily tasks with vigor and safety [28] and with sufficient energy reserves to meet emergencies and/or enjoy leisure or personal development activities [33]. High levels of physical fitness are associated with better physical and cognitive functioning, a better quality of life, and lower health costs [34,35,36].

Sarcopenia has only recently been classified as a medical condition [37], and therefore its importance is still poorly recognized, and diagnosis is scarce in clinical practice. The SARC-F is a simple and easy-to-use screening tool for sarcopenia that would be of great use for identifying sarcopenia in clinical practice. As the pioneer of screening tools for sarcopenia, SARC-F has been widely used in the field of sarcopenia research. The SARC-F has been validated in different ethnic populations [38,39,40,41,42] since it was developed in 2013 [13].

Previous studies have revealed SARC-F to be a valuable tool to predict clinically significant outcomes such as functional impairment, hospitalization [15,16,43,44], poor quality of life, and mortality. Several works that tested SARC-F as a screening tool for sarcopenia consensually reported moderate to high specificity (sp: ~70–90%), that is, the ability to identify elderly people who were not suspected of having sarcopenia and who therefore should not proceed with the diagnostic evaluation [13,45,46,47].

The main limitation of the present study is related to the selection of the reference instrument for the assessment of suspected sarcopenia—the SARC-F—since several screening approaches [48] have been proposed. However, the different sarcopenia screening approaches present validation limitations related to the determination of muscle mass by dual-energy x-ray absorptiometry [49]. Another limitation is that the diagnosis of sarcopenia was not carried out, but only its suspicion through the symptomatology and the analysis of its relationship with physical fitness assessed by the SFT. As strengths of this work, we highlight the recruitment, characterization, and investigation with a peculiar and rarely studied sample, whose participants live in poor and difficult-to-access cities in Brazil where screening is even more important for health promotion and the facilitation of clinical practice.

## 5. Conclusions

The 30-s arm curl test (<11.5 reps) and the 6-min walk test (<408.5 for men and <366.0 m for women) of the SFT showed the ability to discriminate between elderly people from Novo Aripuanã with and without suspicious symptoms of sarcopenia. 

## Figures and Tables

**Table 1:** Descriptive characteristics of the participants: mean ± standard deviation or median (interquartile range) *.

	Mean ± SD			
	All (*n* = 312)	Male (*n* = 112)	Female (*n* = 200)	*p*-Value
Age, years	72.63 ± 7.81	73.07 ± 7.31	72.39 ± 8.09	0.458
Body Height, cm	153.65 ± 8.22	159.99 ± 8.26	150.10 ± 5.67	<0.001
Body Mass, kg	63.70 ± 12.67	69.29 ± 11.61	60.52 ± 12.18	<0.001
BMI, kg/m^2^	26.88 ± 4.65	27.08 ± 4.64	26.76 ± 4.65	0.566
SARC-F score, pts	1.75 ± 1.88	1.43 ± 1.68	1.92 ± 1.95	0.915
SARC-F ≥ 4 pts, *n* (%) ^#^	56 (17.9)	14 (12.5)	42 (21.0)	0.061
Physical Fitness				
Chair Stand Test, *n*	10.86 ± 3.22	11.08 ± 3.34	10.74 ± 3.15	0.365
Arm Curl Test, *n*	12.56 ± 3.83	13.19 ± 4.07	12.22 ± 3.66	0.031
CSAR, cm *	4.00 (11)	6.00 (11)	3.00 (11)	0.284
BST, cm *	−19.00 (21)	−23.00 (18)	−17.00 (23)	<0.001
FUG, seg	8.08 ± 2.67	7.43 ± 2.06	8.44 ± 2.89	<0.001
6MWT, m	407.29 ± 108.43	450.76 ± 125.58	382.95 ± 88.99	<0.001
FAB score, pts	12.44 ± 3.66	13.29 ± 3.07	11.97 ± 3.88	0.002

Notes: SD, standard deviation, BMI, body mass index; SARC-F, sarcopenia screening questionnaire; CSAR, chair sit-and-reach test; BST, back scratch test; FUG, foot up-and-go test; 6MWT, 6-min walk test; FAB, Fullerton Advanced Balance Scale. Comparison between groups using the Chi-square ^#^ or Mann–Whitey test *.

**Table 2:** Prevalence of symptoms of sarcopenia evaluated through the SARC-F questionnaire.

	Male (*n* = 112)			Female (*n* = 200)			
Symptoms	None	Some	A Lot, or Unable	None	Some	A Lot, or Unable	*p*-Value
1. Lack of strength, *n* (%)	79 (70.5)	20 (17.9)	13 (11.6)	133 (66.5)	48 (24.0)	19 (9.5)	0.808
2. Assistance in walking, *n* (%)	93 (83.0)	18 (16.1)	1 (0.9)	156 (78.0)	37 (18.5)	7 (3.5)	0.175
3. Difficulty rising from a chair, *n* (%)	84 (75.0)	26 (23.2)	2 (1.8)	142 (71.0)	55 (27.5)	3 (1.5)	0.521
4. Difficulty climbing stairs, *n* (%)	63 (56.3)	41 (36.6)	8 (7.1)	115 (57.5)	71 (35.5)	14 (7.0)	0.851
5. Falls, *n* (%)	73 (65.2)	39 (34.8)	0 (0.0)	150 (75.0)	50 (25.0)	0 (0.0)	0.124

**Table 3:** Associations through logistic regression between the occurrence of significant symptoms of sarcopenia (≥4 points) based on the participants’ physical fitness.

	SARC-F (Score)							
	Male (*n* = 112)				Female (*n* = 200)			
Functional Fitness Tests	B	*p*	OR	95% CI	B	*p*	OR	95% CI
30-s chair stand test, *n*	−0.244	0.014	0.784	0.645–0.953	−0.105	0.081	0.900	0.800–1.013
30-s arm curl test, *n*	−0.246	0.012	0.782	0.646–0.947	−0.187	0.001	0.829	0.742–0.926
Chair sit-and-reach test, cm	−0.051	0.028	0.950	0.907–0.994	−0.007	0.624	0.993	0.964–1.022
Back scratch test, cm	−0.036	0.126	0.964	0.961–1.010	−0.031	0.026	0.969	0.943–0.996
Foot up-and-go test, seg	0.161	0.182	1.174	0.928–1.486	0.125	0.024	1.133	1.016–1.263
6-min walk test, m	−0.007	0.032	0.993	0.987–0.999	−0.008	0.001	0.992	0.988–0.997
Fullerton Advanced Balance, *n*	−0.084	0.305	0.919	0.783–1.079	−0.095	0.026	0.910	0.837–0.989

CST, 30 s chair stand test. ACT, 30-s arm curl test CSAR, chair sit-and-reach test. BST, back scratch test. FUG, foot up-and-go test. 6MWT, 6-min walk test. 4-MGS, m/s, 4-m gait speed; FAB, Fullerton Advanced Balance Scale. B, betas coefficients.

**Table 4:** Associations through logistic regression between the occurrence of each sarcopenia symptom based on the participants’ physical fitness.

	**Difficulty in Lifting and Carrying 4.5 kg**							
	**Male (*n* = 112)**				**Female (*n* = 200)**			
**Predictor**	**β**	***p***	**OR**	**95% CI**	**β**	***p***	**OR**	**95% CI**
30-s chair stand test, *n*	−0.070	0.276	0.932	0.822–1.058	0.013	0.784	1.013	0.923–1.112
30-s arm curl test, *n*	−0.087	0.128	0.917	0.820–1.025	−0.030	0.476	0.971	0.895–1.053
Chair sit-and-reach test, cm	0.002	0.908	1.002	0.966–1.040	−0.015	0.257	0.985	0.960–1.011
Back scratch test, cm	0.001	0.925	1.001	0.975–1.028	0.022	0.046	1.022	1.000–1.044
8-foot up-and-go test, seg	0.039	0.689	1.040	0.857–1.262	−0.023	0.669	0.978	0.882–1.084
6-min walk test, m	−0.002	0.195	0.998	0.994–1.001	−0.001	0.441	0.999	0.995–1.002
Fullerton Advanced Balance Scale, *n*	−0.069	0.291	0.934	0.822–1.061	0.046	0.258	1.047	0.967–1.132
	**Difficulty in Walking Across a Room**							
	**Male (*n* = 112)**				**Female (*n* = 200)**			
**Predictor**	**β**	***p***	**OR**	**95% CI**	**β**	***p***	**OR**	**95% CI**
30-s chair stand test, *n*	0.008	0.911	1.008	0.870–1.169	-0.037	0.504	0.964	0.864–1.074
30-s arm curl test, *n*	−0.066	0.336	0.936	0.819–1.070	−0.025	0.593	0.975	0.889–1.070
Chair sit-and-reach test, cm	0.005	0.815	1.005	0.961–1.052	−0.025	0.098	0.975	0.947–1.005
Back scratch test, cm	−0.024	0.215	0.977	0.941–1.014	0.015	0.218	1.015	0.991–1.039
8-foot up-and-go test, seg	−0.015	0.904	0.985	0.772–1.257	0.012	0.837	1.012	0.903–1.134
6-min walk test, m	0.001	0.956	1.000	0.996–1.004	0.001	0.730	1.001	0.997–1.004
Fullerton Advanced Balance Scale, *n*	−0.036	0.645	0.965	0.828–1.124	0.033	0.467	1.034	0.945–1.131
	**Difficulty in Transferring from a Chair or Bed**							
	**Male (*n* = 112)**				**Female (*n* = 200)**			
**Predictor**	**β**	***p***	**OR**	**95% CI**	**β**	***p***	**OR**	**95% CI**
30 s chair stand test, *n*	−0.031	0.635	0.696	0.851–1.103	−0.098	0.065	0.907	0.817–1.006
30 s arm curl test, *n*	−0.012	0.819	0.988	0.887–1.099	−0.101	0.027	0.904	0.827–0.989
Chair sit-and-reach test, cm	0.007	0.728	1.007	0.968–1.047	−0.022	0.109	0.978	0.952–1.005
Back scratch test, cm	−0.016	0.299	0.984	0.955–1.014	0.018	0.103	1.018	0.996–1.041
8-foot up-and-go test, seg	−0.090	0.435	0.914	0.729–1.146	0.013	0.808	1.013	0.913–1.125
6-min walk test, m	−0.001	0.655	0.999	0.996–1.003	−0.001	0.672	0.999	0.996–1.003
Fullerton Advanced Balance Scale, *n*	0.086	0.297	1.090	0927–1.280	0.049	0.245	1.050	0.967–1.141
	**Difficulty in Climbing a Flight of 10 Stairs**							
	**Male (*n* = 112)**				**Female (*n* = 200)**			
**Predictor**	**β**	***p***	**OR**	**95% CI**	**β**	***p***	**OR**	**95% CI**
30-s chair stand test, *n*	−0.023	0.962	0.977	0.873–1.094	−0.036	0.428	0.964	0.881–1.055
30-s arm curl test, *n*	−0.050	0.301	0.951	0.864–1.046	−0.003	0.929	0.997	0.923–1.076
Chair sit-and-reach test, cm	−0.014	0.414	0.986	0.953–1.020	0.007	0.581	1.007	0.983–1.032
Back scratch test, cm	0.002	0.841	1.002	0.978–1.027	0.018	0.091	1.018	0.997–1.039
8-foot up-and-go test, seg	0.005	0.957	1.005	0.838–1.205	0.005	0.917	1.005	0.912–1.107
6-min walk test, m	−0.001	0.394	0.999	0.996–1.002	0.001	0.868	1.000	0.997–1.003
Fullerton Advanced Balance Scale, *n*	−0.084	0.188	0.919	0.811–1.042	0.029	0.439	1.029	0.957–1.108
	**Falls in the Past Year**							
	**Male (*n* = 112)**				**Female (*n* = 200)**			
**Predictor**	**β**	***p***	**OR**	**95% CI**	**β**	***p***	**OR**	**95% CI**
30-s chair stand test, *n*	0.049	0.411	1.051	0.934–1.182	0.061	0.230	1.063	0.962–1.175
30-s arm curl test, *n*	−0.043	0.399	0.958	0.867–1.059	0.001	0.991	1.000	0.917–1.092
Chair sit-and-reach test, cm	0.016	0.374	1.017	0.980–1.054	0.016	0.276	1.016	0.988–1.044
Back scratch test, cm	−0.012	0.371	0.988	0.962–1.015	−0.019	0.127	0.981	0.958–1.005
8-foot up-and-go test, seg	−0.076	0.453	0.927	0.759–1.131	0.032	0.564	1.032	0.927–1.149
6-min walk test, m	0.001	0.786	1.000	0.996–1.003	−0.002	0.245	0.998	0.994–1.002
Fullerton Advanced Balance Scale, *n*	0.033	0.626	1.033	0.906–1.179	0.014	0.043	0.107	0.933–1.103

## Data Availability

The data presented in this study are available on request from the corresponding author. The data are not publicly available as they belong to a database of a Ph.D. thesis in progress.
